# Common Infections in Patients Prescribed Systemic Glucocorticoids in Primary Care: A Population-Based Cohort Study

**DOI:** 10.1371/journal.pmed.1002024

**Published:** 2016-05-24

**Authors:** Laurence Fardet, Irene Petersen, Irwin Nazareth

**Affiliations:** 1 Department of Primary Care and Population Health, University College London, London, United Kingdom; 2 Department of Dermatology, Henri Mondor Hospital, Paris, France; 3 EA 7379 Epidémiologie en Dermatologie et Evaluation des Thérapeutiques, Université Paris–Est Créteil, UPEC Paris 12, Créteil, France; University of Oxford, UNITED KINGDOM

## Abstract

**Background:**

Little is known about the relative risk of common bacterial, viral, fungal, and parasitic infections in the general population of individuals exposed to systemic glucocorticoids, or about the impact of glucocorticoid exposure duration and predisposing factors on this risk.

**Methods and Findings:**

The hazard ratios of various common infections were assessed in 275,072 adults prescribed glucocorticoids orally for ≥15 d (women: 57.8%, median age: 63 [interquartile range 48–73] y) in comparison to those not prescribed glucocorticoids. For each infection, incidence rate ratios were calculated for five durations of exposure (ranging from 15–30 d to >12 mo), and risk factors were assessed. Data were extracted from The Health Improvement Network (THIN) primary care database. When compared to those with the same underlying disease but not exposed to glucocorticoids, the adjusted hazard ratios for infections with significantly higher risk in the glucocorticoid-exposed population ranged from 2.01 (95% CI 1.83–2.19; *p <* 0.001) for cutaneous cellulitis to 5.84 (95% CI 5.61–6.08; *p <* 0.001) for lower respiratory tract infection (LRTI). There was no difference in the risk of scabies, dermatophytosis and varicella. The relative increase in risk was stable over the durations of exposure, except for LRTI and local candidiasis, for which it was much higher during the first weeks of exposure. The risks of infection increased with age and were higher in those with diabetes, in those prescribed higher glucocorticoid doses, and in those with lower plasma albumin level. Most associations were also dependent on the underlying disease. A sensitivity analysis conducted on all individuals except those with asthma or chronic obstructive pulmonary disease produced similar results. Another sensitivity analysis assessing the impact of potential unmeasured confounders such as disease severity or concomitant prescription of chemotherapy suggested that it was unlikely that adjusting for these potential confounders would have radically changed the findings. Limitations of our study include the use of electronic medical records, which could have resulted in some degree of misclassification of the infectious outcomes; a possible reporting bias, as general practitioners could be more prone to record an infection in those exposed to glucocorticoids; and a low number of events for some outcomes such as scabies or varicella, which may have led to limited statistical power.

**Conclusions:**

The relative risk of LRTI and local candidiasis is very high during the first weeks of glucocorticoid exposure. Further studies are needed to assess whether low albumin level is a risk factor for infection by itself (e.g., by being associated with a higher free glucocorticoid fraction) or whether it reflects other underlying causes of general debilitation.

## Abstract 2

Fardet and colleagues present a large population study assessing the risks of developing infections following glucocorticoid treatment. They describe the impact of age and other conditions such as diabetes on developing infections.

## Editors’ Summary

**Background:**

Throughout life, our immune system protects us from attack by disease-causing organisms. When a virus, bacterium, fungus, or parasite enters the human body, the immune system detects the invader and triggers a response that kills or neutralizes it. Normally, the immune system discriminates perfectly between foreign invaders and the body’s own cells and tissues, but sometimes it goes awry and begins to attack “self,” resulting in the development of an autoimmune disease such as rheumatoid arthritis (in which the immune system attacks the joints) or lupus erythematosus (in which the immune system attacks numerous tissues and organs). Immune system dysfunction is also involved in chronic inflammatory conditions. Tissue inflammation (pain, heat, redness, and swelling) is part of the normal immune response, but uncontrolled inflammation underlies sepsis (a systemic inflammatory condition that sometimes develops after a localized infection) and lung conditions such as asthma and chronic obstructive pulmonary disease.

**Why Was This Study Done?:**

Inflammatory diseases and autoimmune diseases are often treated with glucocorticoids—drugs with immunosuppressive and anti-inflammatory properties. At any one time, more than 1% of the US and UK population is receiving systemic glucocorticoid therapy (whole-body therapy often given as a pill) to treat an autoimmune or inflammatory condition or to treat cancer or prevent organ rejection after transplantation. But, although prednisolone, dexamethasone, and other glucocorticoids are effective treatments for these conditions, long-term use of oral glucocorticoids is associated with serious side effects, including an increased infection risk. Several studies have investigated the overall risk of infection in specific populations receiving glucocorticoids, but little is known about the risk of individual common infections among people in the general population taking glucocorticoids. Here, the researchers undertake a population-based cohort study—an observational investigation that compares specific outcomes in groups (cohorts) of people from the general population with different baseline characteristics—to discover more about common infections in patients prescribed systemic glucocorticoids in primary care.

**What Did the Researchers Do and Find?:**

Using The Health Improvement Network (THIN) primary care database, the researchers identified 275,072 adults prescribed oral glucocorticoids in the UK between 2000 and 2012, a random sample of people not prescribed glucocorticoids but with a diagnosis of the same underlying disease as the exposed individuals, and cases of infection in both cohorts. Compared to the unexposed group, the glucocorticoid-exposed group had an increased risk of three bacterial infections (septicemia, lower respiratory tract infection [LRTI], and cutaneous cellulitis), one viral infection (herpes zoster), and one fungal infection (local candidiasis) but no increased risk of varicella virus infection, dermatophytosis (a fungal infection), or scabies (a parasitic infection). The increased risk was highest for LRTI—the risk of LRTI was nearly six-fold higher in the glucocorticoid-exposed group than in the unexposed group. The relative increase in infection risk was similar whatever the duration of glucocorticoid therapy except for LRTI and local candidiasis, for which it was higher in the first few weeks of treatment. The risk of infection increased with age and was higher in people with diabetes, in people given high glucocorticoid doses, and in people with low blood albumin levels. Finally, the risks of different infections depended on the disease for which glucocorticoids were prescribed.

**What Do These Findings Mean?:**

These findings show that the risk of several (but not all) common bacterial, viral, and fungal infections was higher among people prescribed glucocorticoids for a range of underlying diseases than among people matched for age, gender, and underlying disease who were not prescribed glucocorticoids. Notably, the relative risk of LRTI and local candidiasis was particularly high during the first weeks of glucocorticoid exposure. Several aspects of the study design may affect the accuracy of these findings. For example, the use of medical records may have resulted in some misclassification of infectious outcomes. However, the finding of a high risk of LRTI in patients prescribed systemic glucocorticoids should be considered before prescribing these drugs, particularly in patients with asthma or chronic obstructive pulmonary disease. The finding of an association between low blood albumin level and increased infection risk needs further investigation to determine whether a low albumin level is a direct risk factor for infection or a marker for other underlying causes of debilitation that increase the risk of infection, but it may be worth considering monitoring albumin level before and during glucocorticoid treatment.

**Additional Information:**

This list of resources contains links that can be accessed when viewing the PDF on a device or via the online version of the article at http://dx.doi.org/10.1371/journal.pmed.1002024.

- This study is further discussed in a *PLOS Medicine* Perspective by Lionel Rostaing and Paolo Malvezzi
- The UK National Health Service Choices website provides information about corticosteroids (glucocorticoids are a specific type of corticosteroids)
- The Mayo Clinic has an article about the benefits and risks of corticosteroids
- Wikipedia has information on autoimmune disease, inflammatory disorders, and glucocorticoids (note that Wikipedia is a free online encyclopedia that anyone can edit; available in several languages)
- MedlinePlus provides links to further information about autoimmune diseases and about corticosteroids
- More information about The Health Improvement Network database of anonymized electronic medical records is available

## Introduction

More than 1% of the general population in the US and the UK receives systemic glucocorticoid therapy, and this figure has increased by more than 30% over the last 20 y [1,2]. Many patients are exposed to glucocorticoids for many weeks or months in primary care [1,2], especially for respiratory (e.g., asthma), rheumatic (e.g., giant cell arteritis, rheumatoid arthritis), or neoplastic conditions [1]. Even though the efficacy of glucocorticoids in the treatment of these conditions is indisputable, they can be associated with severe adverse events. Infections are known complications of systemic glucocorticoid exposure, even in those exposed for only a few days or weeks [3]. A meta-analysis of randomized controlled trials (RCTs) published more than 25 y ago found that the overall risk of infections was 50% to 60% higher in the glucocorticoid-exposed population compared to those receiving placebo [3]. The increase in risk is much higher for opportunistic infections (e.g., tuberculosis, listeriosis, invasive fungal infections) and in specific populations (e.g., allogeneic bone marrow transplant, solid organ transplant) [4–10]. Surprisingly, very few studies have focused on the risk of various common infectious conditions in the general population of glucocorticoid-treated patients, and it is unclear whether there is a differential risk regarding bacterial, viral, fungal, and parasitic infections. Moreover, because previous studies were conducted in selected populations and used different methods, the impact of the underlying condition on the risk of infection is difficult to assess. Further, limited evidence is available regarding predisposing factors that may contribute to an increased risk of infection in individuals.

In this study, we aimed to (1) assess the relative risks of various common bacterial, viral, parasitic, and fungal infections in people prescribed systemic glucocorticoids in primary care, (2) compare these relative risks between different durations of glucocorticoid exposure, and (3) identify clinical and biological factors associated with the risk of infectious events in individuals prescribed systemic glucocorticoids.

## Methods

All methods were prespecified unless otherwise noted.

### Ethics

The Health Improvement Network (THIN) scheme for obtaining patient data and providing them in anonymized form to researchers was approved by the National Health Service South-East Multicentre Research Ethics Committee in 2002. The present study was approved by the University College London THIN steering committee and by the THIN scientific review committee (number 14–072).

### Data Source: The Health Improvement Network

Approximately 98% of the population in the UK is registered with a general practitioner. THIN is a database of anonymized electronic medical records from UK general practices. Participating general practitioners systematically and prospectively retrieve and enter clinical information on patients, including demographics data, diagnoses, and prescriptions, so that the database provides a longitudinal medical record for each patient. THIN is representative of the UK population, and comparisons to external statistics and other independent studies have shown that both the clinical diagnostic and prescribing information is well recorded and accurate [11,12]. The data are entered in routine general practice and therefore reflect “real life” clinical care. Prescribing is well recorded in terms of general practitioner prescriptions since the computerized entry made by the doctor is also used to issue a prescription to the patient. To date, THIN includes data from almost 600 general practices and more than 12 million individuals. For this study, we used data from 1 January 2000 to 31 December 2012 from all general practices that contributed to the database during this period. The data used for this study were obtained from a license to THIN. For further information on access to the database, please contact IMS Health (contact details can be found at http://www.csdmruk.imshealth.com/index.html).

### Identification of Glucocorticoid Prescriptions

In THIN, each prescription of a drug is recorded as a code referenced to the relevant chapter in the British National Formulary [13]. We selected all synthetic glucocorticoids prescribed orally, and this included prednisolone, prednisone, dexamethasone, triamcinolone, betamethasone, methylprednisolone and deflazacort. In the case of multiple consecutive prescriptions, we considered that the prescriptions were part of a single course of therapy if the previous prescription was issued less than 1 mo earlier, in order to take into account the persistence of glucocorticoid action on immunity during the days following stopping glucocorticoid use. The start of a course was defined as the day of the first prescription. The end of a course was defined as the last day of the last prescription. These dates were derived from two variables available in the database: total number of pills prescribed and number of pills to be taken per day. The baseline daily dosage was calculated from the first glucocorticoid prescription. It was derived from the number of pills prescribed per day multiplied by the dosage of each pill calculated in prednisone equivalent.

### Identification of Infectious Events

All diagnoses and symptoms are recorded in THIN using the Read classification system [14]. This classification was used to create medical lists that enabled us to identify cases of three bacterial (i.e., septicemia, lower respiratory tract infection [LRTI], cutaneous cellulitis), two viral (i.e., herpes zoster, varicella), one parasitic (i.e., scabies), and two fungal (i.e., local candidiasis, dermatophytosis) infections recorded in the database (code lists available in S1 Table). These infections were chosen because they cover a set of bacterial/viral/fungal/parasitic and local/systemic infections frequently diagnosed in primary care. We initially planned to include infectious colitis based on previous work suggesting an association between infectious colitis and glucocorticoid exposure, but exploratory work indicated that this infection was poorly recorded in primary care. In order to ensure that we identified true index cases, we chose to be specific rather than sensitive in choosing the codes relevant to infections. We restricted our choice to those infections for which there was a precise code. Hence, for septicemia, we restricted the codes to bacterial septicemia. For instance, those relating to “herpes simplex septicemia” or “candidal septicemia” were excluded. Likewise, the codes selected for bacterial LRTI were specific to bacterial agents, and codes such as “viral pneumonia” or “rheumatic pneumonia” or value codes such as “chest infection” or “bronchitis” were excluded. Finally, the codes selected for cutaneous cellulitis were again specific to the diagnosis, and imprecise codes such as “bacterial skin infections” or “fasciitis unspecified” were excluded (see S1 Table). In cases of several records of the same type (e.g., recurrent herpes zoster or several records for an episode of chronic local candidiasis or dermatophytosis) during the glucocorticoid exposure period (for exposed individuals) or the at-risk period (for unexposed individuals; see below), the date of the first record was used. Lastly, all patients with an infection recorded within the first 15 d of glucocorticoid initiation were excluded from the analyses, as the symptoms associated with the diagnosed infection may have been the reason for prescribing the glucocorticoid rather than the infection being the consequence of glucocorticoid exposure (i.e., protopathic bias). We did the same for the unexposed populations and thus excluded from the analyses all patients with an infectious event recorded within 15 d after the randomly selected “index date.”

### Glucocorticoid-Exposed Group

We identified all adults who were prescribed at least one course of oral glucocorticoid for at least 15 d. As it is likely that those who have had a severe infection in the past while being treated with glucocorticoids are less likely to be re-prescribed glucocorticoids, we chose to include only people at their first glucocorticoid exposure. As events recorded within the first 6 mo of registration are more likely to represent retrospective recording of a past history rather than a new episode of a problem, we selected only people who started glucocorticoids at least 6 mo after their registration in order to ensure capturing people with incident rather than prevalent prescriptions. The medical diagnosis recorded on the date of starting glucocorticoids was used as the indication for the glucocorticoid prescription. If there was no medical diagnosis recorded on this date, we searched for seven relevant conditions (i.e., asthma, chronic obstructive pulmonary disease [COPD], rheumatoid arthritis, inflammatory bowel disease, polymyalgia rheumatica/giant cell arteritis, connective tissue disease [lupus erythematosus, dermatomyositis, polymyositis, systemic sclerosis or undifferentiated connective tissue disease], and cancer) entered in the records up to 1 y prior to or after this prescription. For those with two (or more) of these conditions (e.g., COPD and cancer) recorded in the medical file, we took into account the condition recorded closest to when the glucocorticoid prescription was issued.

### Unexposed Groups

Two comparison groups were identified. The first was a random sample of people who were not prescribed glucocorticoids (unexposed population #1), and the second was a random sample of people not prescribed glucocorticoids but with a diagnosis of the same underlying disease of interest as the exposed patients (unexposed population #2). People exposed to glucocorticoids for any duration of exposure at any time after registration in THIN were excluded from the control populations. We selected up to three unexposed individuals from each unexposed group (without and with the same underlying disease of interest) for every exposed individual. When selecting the unexposed groups, we stratified the samples in terms of sex and age (within 10-y age bands) to ensure the distribution of these groups was similar to that of the glucocorticoid-exposed group. For each unexposed individual, a randomly selected “index date” was defined at least 6 mo after their registration. This “index date” was defined as the start of the at-risk period. The end of the at-risk period was also randomly defined, at least 15 d after the “index date,” so that the at-risk period of the unexposed individuals was similar to the exposure period of the exposed individuals.

### Biological Data

We searched the medical file of all glucocorticoid-exposed patients included in the study in order to extract data regarding lymphocyte count and albumin level measured before record of the infectious event (or before a randomly defined date during exposure for those who did not have any of the infections of interest recorded). After exploratory work, we chose to include in the analyses biological data recorded within the period ranging from 3 mo to 1 d before these dates. In cases of several available lymphocyte counts and albumin levels during the period of interest, means were calculated and used in the analyses.

### Statistical Analysis

For each participant, follow-up time (i.e., exposure or at-risk period) was accrued from 15 d after the glucocorticoid start date (in the exposed population) or the index date (in the unexposed population) until the date of the infectious outcome, the end of the at-risk period, the date of leaving the practice, the date of death, or the end of the study period, whichever occurred first. First, we compared the exposed to the unexposed groups to assess the hazard ratios of infections associated with the prescription of glucocorticoids using Cox proportional hazards models adjusted for age, sex, use of another immunosuppressant (i.e., methotrexate, azathioprine, cyclosporine, or mycophenolate mofetil; coded as a binary variable: “0” if no use of another immunosuppressant during the exposure/at-risk period and “1” if at least one prescription of any immunosuppressant during this time period), past medical history of diabetes, and, for the comparison with the second unexposed group, the underlying disease. Second, for each first episode of infection, standardized incidence rate ratios were calculated during five durations of exposure (i.e., 15–30 d, 1–3 mo, 4–6 mo, 7–12 mo, >12 mo) in order to assess whether there was differential risk according to the duration of exposure. Lastly, risk factors for infections in those exposed to systemic glucocorticoids were assessed using Cox proportional hazards models comparing those with an infectious outcome during glucocorticoid exposure to those without.

We further conducted three types of sensitivity analyses. In the first sensitivity analysis, we excluded from the analyses all patients with asthma or COPD in order to ensure that the relative risk variations over duration of exposure were not related to the high proportion of people with these two conditions in the study population. In the second sensitivity analysis, we assessed risk factors for infection in those exposed to glucocorticoids after excluding all infections recorded within 1 mo after glucocorticoid initiation rather than 15 d. In the third sensitivity analysis, because we found that the underlying condition could be strongly associated with the risk of some infectious outcomes (e.g., asthma/COPD with LRTI, cancer with septicemia), we tried to estimate the impact of some unmeasured confounders, such as severity of the disease being treated or other concomitant prescriptions such as chemotherapy, on these findings by using a Schneeweiss rule-out approach [15]. The proportional hazards assumption for Cox models was checked graphically using the Schoenfeld residuals. No interaction term was included in the models. Continuous variables are presented as median (interquartile range [IQR]). Categorical variables are presented as proportions. The groups’ characteristics were compared using the Chi^2^ test for comparisons of proportions and the Wilcoxon rank-sum test for comparisons of medians. All statistical tests were two-sided. A *p*-value of <0.05 was considered statistically significant. All analyses were done using Stata, version 11.2, except for the Schneeweiss rule-out approach, which was performed in Excel as described by Sebastian Schneeweiss [15,16].

## Results

### Study Populations

**Fig 1:**
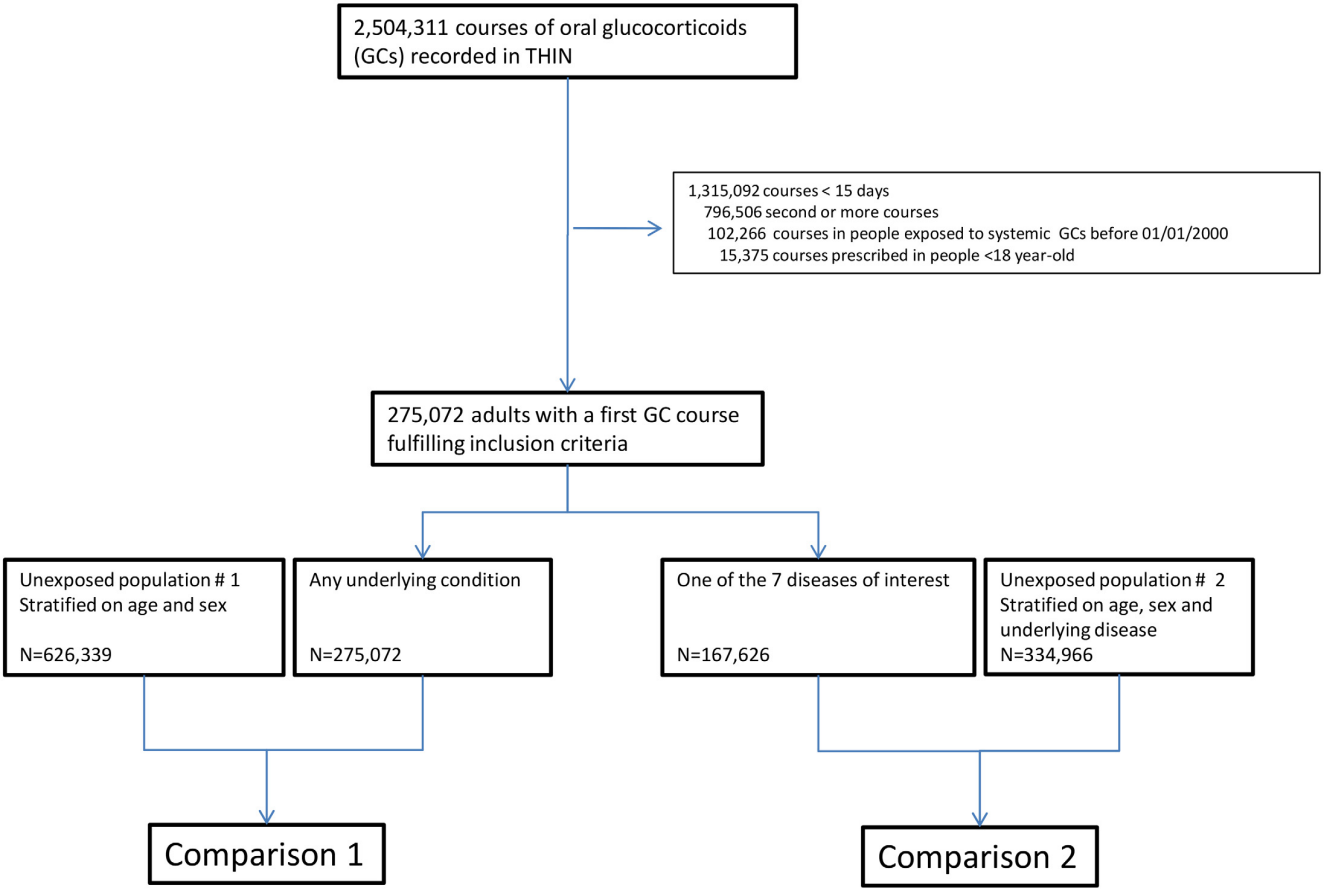
Study flowchart.

In total, 275,072 adults were prescribed at least one course of oral glucocorticoids for ≥15 d during the study period (women: 57.8%, median age: 63 [IQR 48–73] y). For 167,626 (60.9%) of them (women: 57.8%, median age: 65 [IQR 50–75] y), glucocorticoids were prescribed for one of the seven diseases of interest, i.e., asthma, COPD, rheumatoid arthritis, inflammatory bowel disease, polymyalgia rheumatica/giant cell arteritis, cancer, or connective tissue disease (Fig 1). The characteristics of both the exposed and the unexposed populations are reported in Table 1. Those prescribed glucocorticoids were more likely to have a past history of diabetes and were much more frequently exposed to other immunosuppressants than those not prescribed glucocorticoids (Table 1).

**Table 1:** Study populations.

Characteristic	All GC-Exposed Patients (*n* = 275,072)	Unexposed Population #1 (Stratified on Age and Sex) (*n* = 626,339)	*p*-Value	Patients Exposed to GC for One of the Seven Diseases of Interest (*n* = 167,626)	Unexposed Population #2 (Stratified on Age, Sex, and Underlying Disease) (*n* = 334,966)	*p*-Value
**Age, years** *	63 (48–73)	62 (46–74)		65 (50–75)	61 (46–74)	
**Women** *	158,999 (57.8%)	363,290 (58.0%)		96,892 (57.8%)	187,709 (56.0%)	
**Start/index date**	Apr 2007 (Jan 2004–Apr 2010)	Jun 2007 (Mar 2004–Sep 2010)	<0.001	Apr 2007 (Feb 2004–May 2010)	May 2007 (Aug 2003–Sep 2010)	<0.001
**Underlying disease**						<0.001
Asthma	—	—		62,163 (37.1%)	155,224 (46.4%)	
COPD	—	—		34,995 (20.9%)	56,642 (16.9%)	
Cancer	—	—		26,502 (15.8%)	78,745 (23.5%)	
Polymyalgia rheumatica/giant cell arteritis	—	—		23,254 (13.9%)	5,804 (1.7%)	
Inflammatory bowel disease	—	—		9,614 (5.7%)	15,867 (4.7%)	
Rheumatoid arthritis	—	—		7,006 (4.2%)	14,169 (4.2%)	
Connective tissue disease	—	—		4,092 (2.4%)	8,515 (2.6%)	
**Past history of diabetes**	40,551 (14.7%)	67,136 (10.7%)	<0.001	24,986 (14.9%)	41,956 (12.5%)	<0.001
**Duration of GC exposure, days**						
Median, days	33 (21–70)	—		36 (22–77)	—	
15–30 d	129,292 (47.0%)	—		74,296 (44.3%)	—	
1–3 mo	90,945 (33.1%)	—		56,863 (33.9%)	—	
4–6 mo	26,728 (9.7%)	—		17,187 (10.3%)	—	
7–12 mo	16,460 (6.0%)	—		11,309 (6.7%)	—	
>12 mo	11,647 (4.2%)	—		7,971 (4.8%)	—	
**Initial GC daily dosage** **						
Median, mg	15 (10–30)	—		20 (10–30)	—	
<20 mg	140,031 (50.9%)	—		81,481 (48.6%)	—	
20–50 mg	123,478 (44.9%)	—		78,677 (46.9%)	—	
>50 mg	11,5363 (4.2%)	—		7,468 (4.5%)	—	
**Other immunosuppressants** ***			<0.001			<0.001
Methotrexate	4,406 (1.6%)	748 (0.1%)		3,104 (1.9%)	3,031 (0.9%)	
Azathioprine	3,993 (1.5%)	101 (<0.1%)		2,203 (1.3%)	537 (0.2%)	
Cyclosporine	760 (0.3%)	57 (<0.1%)		161 (0.1%)	50 (<0.1%)	
Mycophenolate mofetil	907 (0.3%)	24 (<0.1%)		239 (0.1%)	21 (<0.1%)	

Data are given as median (IQR) or *n* (percent).

*Unexposed populations stratified on these variables.

**Prednisone equivalent.

***During exposure/at-risk period.

GC, glucocorticoid.

### Risk of Infection

**Fig 2:**
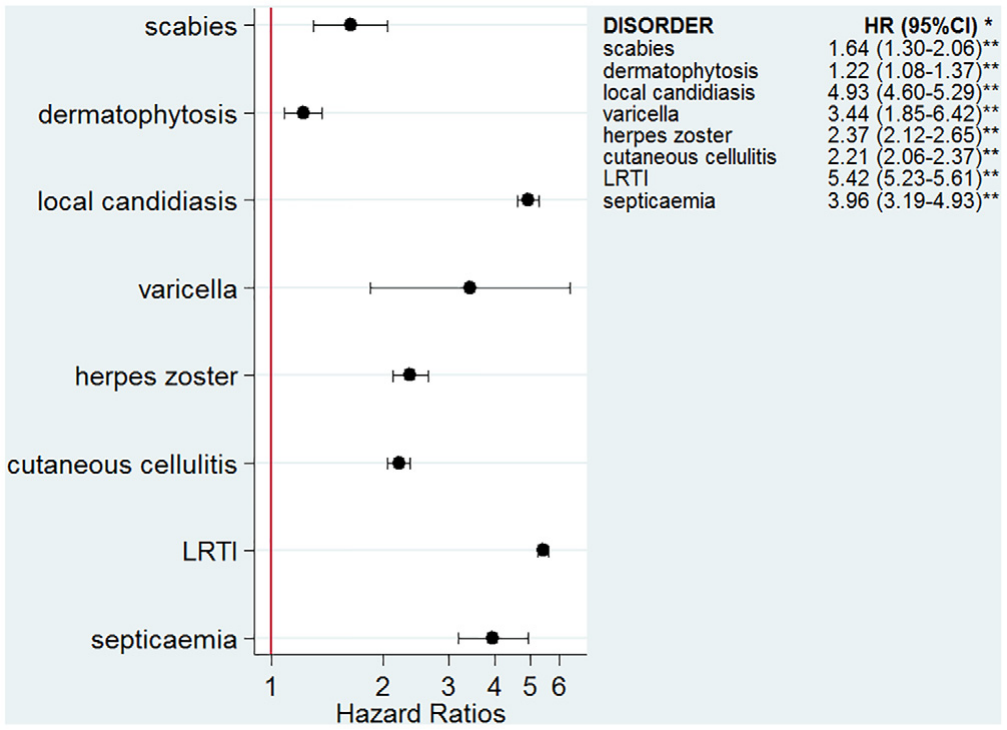
Risk of infection in the glucocorticoid-exposed group compared to those unexposed to glucocorticoids. *Models adjusted for sex, age, diabetes, and use of other immunosuppressants. **p-Value < 0.001 for all infections except for dermatophytosis, *p* = 0.001.

**Fig 3:**
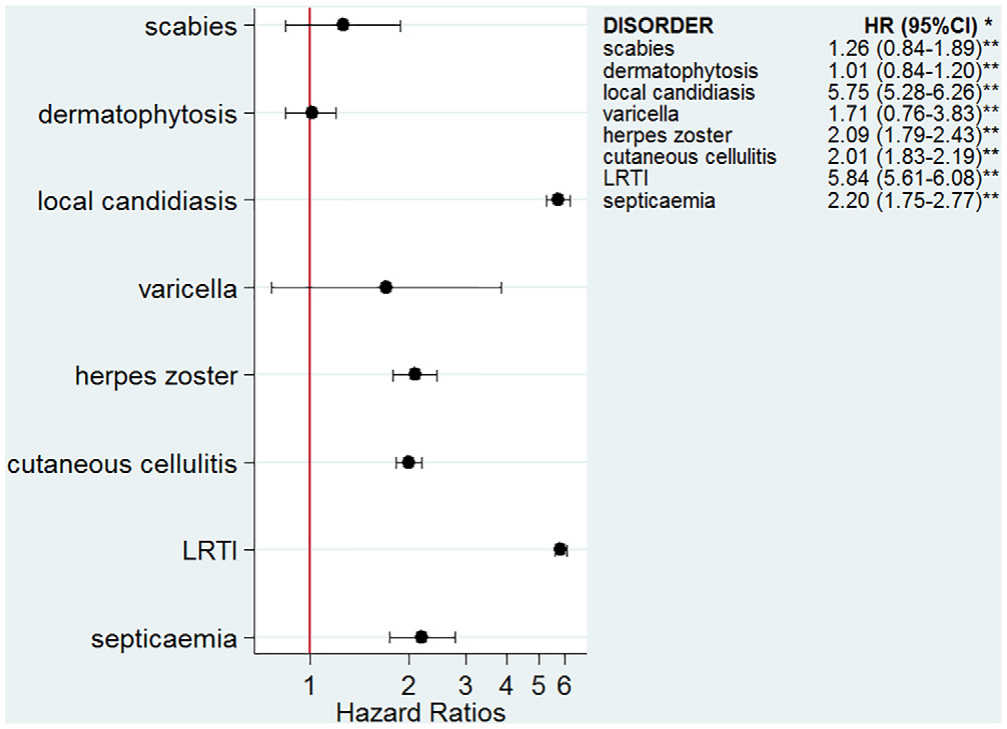
Risk of infection in glucocorticoid-exposed patients with one of the seven diseases of interest compared to those with the same underlying diseases but unexposed to glucocorticoids. *Models adjusted for sex, age, diabetes, use of other immunosuppressants, and the underlying disease. **p-Value < 0.001 for all infections except for scabies, *p* = 0.25; varicella, *p* = 0.20; and dermatophytosis, *p* = 0.97.

The numbers of infectious outcomes of interest are reported in Table 2. The most frequently recorded infectious event both in the exposed and unexposed populations was LRTI (4.3% of all glucocorticoid-exposed patients compared to 0.7% in the unexposed patients), while the least frequently recorded were varicella and scabies (less than 0.1% in both exposed and unexposed individuals). The adjusted hazard ratios are reported in Fig 2. The risk of each infection was increased in the overall population of glucocorticoid-exposed individuals compared to those not exposed to glucocorticoids (Fig 2), with adjusted hazard ratios ranging from 1.22 (95% CI 1.08–1.37; *p* = 0.001) for dermatophytosis to 5.42 (95% CI 5.23–5.61; *p <* 0.001) for LRTI. When people prescribed systemic glucocorticoids for one of the seven diseases of interest were compared to those with the same underlying disease but unexposed to glucocorticoids (Fig 3), we still observed large effects for LRTI and local candidiasis (adjusted hazard ratio 5.84 [95% CI 5.61–6.08] and 5.75 [95% CI 5.28–6.26], respectively; *p <* 0.001). For other infections, the increased risk was lower, around 2-fold, and there was no difference in the risk of scabies (*p* = 0.25), dermatophytosis (*p* = 0.97), and varicella (*p* = 0.20).

**Table 2:** Infectious events recorded during the exposure/at-risk period.

Variable	All GC-Exposed Patients (*n* = 275,072)	Unexposed Population #1 (Stratified on Age and Sex) (*n* = 626,339)	Patients Exposed to GC for One of the Seven Diseases of Interest (*n* = 167,626)	Unexposed Population #2 (Stratified on Age, Sex, and Underlying Disease) (*n* = 334,966)
**Total exposure/at-risk time, person-years**	64,240	136,870	41,687	80,210
**Infection, *n* (percent)**				
Septicemia	283 (0.1%)	133 (<0.05%)	185 (0.1%)	173 (0.5%)
LRTI	11,756 (4.3%)	4,662 (0.7%)	8,625 (7.6%)	4,432 (1.3%)
Cutaneous cellulitis	2,149 (0.8%)	1,604 (0.3%)	1,392 (0.8%)	1,235 (0.4%)
Herpes zoster	837 (0.3%)	640 (0.1%)	555 (0.3%)	453 (0.1%)
Varicella	24 (<0.05%)	23 (<0.05%)	13 (<0.05%)	26 (<0.05%)
Local candidiasis	2,592 (0.9%)	1,304 (0.2%)	1,857 (1.1%)	998 (0.3%)
Dermatophytosis	510 (0.2%)	882 (0.1%)	322 (0.2%)	584 (0.2%)
Scabies	147 (0.1%)	177 (<0.05%)	58 (<0.05%)	95 (<0.05%)

GC, glucocorticoid.

### Relative Increase of Risk with Duration of Exposure

**Fig 4:**
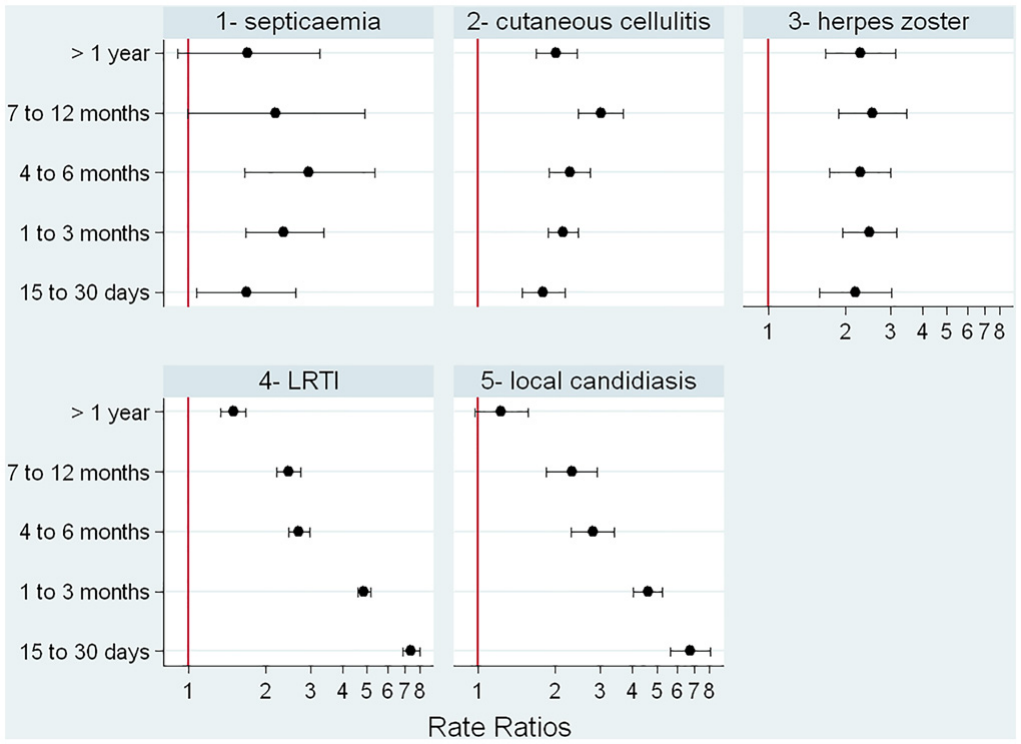
Incidence rate ratios according to duration of glucocorticoid exposure.

When those exposed to glucocorticoids were compared to those with the same underlying conditions but not exposed to glucocorticoids, we found that relative risk of a first episode of each infection varied according to the type of infection (Fig 4). The relative increase in risk was quite stable over duration of exposure for herpes zoster, septicemia, and cutaneous cellulitis. For LRTI and local candidiasis, risk was highest during the first weeks of exposure and markedly decreased thereafter.

### Clinical Risk Factors for Infection in Those Exposed to Glucocorticoids

In order to assess factors associated with the risk of infection in the glucocorticoid-exposed population, those with an infectious outcome during glucocorticoid exposure were compared to those for whom no infectious outcome was recorded during glucocorticoid exposure. We found that the risk of infection increased with age and was higher in those with diabetes and in those prescribed a higher dosage of glucocorticoids (Table 3). The underlying condition was also strongly associated with the infectious outcome. For instance, people with asthma or COPD were at higher risk of LRTI, and those with cancer were at higher risk of septicemia or local candidiasis. Overall, patients with polymyalgia rheumatica/giant cell arteritis were at lower relative risk of infection than those with another underlying disease. Interestingly, the risk of infectious event was not increased by a concomitant prescription of another immunosuppressant, except for herpes zoster.

**Table 3:** Risk factors for infection in the overall population exposed to systemic glucocorticoids (*n* = 275,072).

Variable	Infectious Outcome									
	Septicemia (*n* = 283)		LRTI (*n* = 11,756)		Cutaneous Cellulitis (*n* = 2,149)		Herpes Zoster (*n* = 837)		Local Candidiasis (*n* = 2,592)	
	HR (95% CI)	*p*-Value	HR (95% CI)	*p*-Value	HR (95% CI)	*p*-Value	HR (95% CI)	*p*-Value	HR (95% CI)	*p*-Value
**Age, per 10-y increase**	1.12 (1.03, 1.22)	0.006	1.06 (1.05, 1.07)	<0.001	1.38 (1.34, 1.43)	<0.001	1.10 (1.04, 1.15)	<0.001	0.92 (0.89, 0.94)	<0.001
**Gender, women versus men**	0.89 (0.71, 1.12)	0.31	1.04 (1.00, 1.07)	0.07	1.35 (1.23, 1.48)	<0.001	0.97 (0.84, 1.12)	0.69	1.51 (1.39, 1.64)	<0.001
**Underlying disease**										
Asthma	1	—	1	—	1	—	1	—	1	—
COPD	2.90 (1.38, 6.08)	0.005	1.17 (1.10, 1.23)	<0.001	1.06 (0.88, 1.29)	0.52	1.14 (0.73, 1.49)	0.83	1.00 (0.85, 1.17)	0.98
RA	2.53 (1.09, 5.90)	0.03	0.32 (0.29, 0.36)	<0.001	0.81 (0.65, 1.02)	0.07	1.04 (0.71, 1.51)	0.84	0.46 (0.36, 0.58)	<0.001
IBD	2.85 (1.08, 7.53)	0.03	0.17 (0.15, 0.21)	<0.001	0.76 (0.54, 1.09)	0.14	0.82 (0.47, 1.43)	0.49	0.55 (0.43, 0.71)	<0.001
PMR/GCA	1.20 (0.58, 2.49)	0.62	0.19 (0.17, 0.20)	<0.001	0.50 (0.42, 0.59)	<0.001	0.99 (0.74, 1.32)	0.93	0.34 (0.29, 0.40)	<0.001
Cancer	11.15 (5.78, 21.53)	<0.001	0.63 (0.59, 0.67)	<0.001	1.06 (0.88, 1.28)	0.55	1.76 (1.28, 2.40)	<0.001	2.07 (1.82, 2.35)	<0.001
CTD	4.51 (1.89, 10.75)	0.001	0.28 (0.24, 0.33)	<0.001	1.02 (0.77, 1.35)	0.89	1.66 (1.11, 2.49)	0.01	0.68 (0.53, 0.88)	0.003
Other	3.11 (1.62, 5.99)	0.001	0.35 (0.34, 0.37)	<0.001	0.82 (0.70, 0.95)	0.01	1.10 (0.84, 1.45)	0.48	0.59 (0.52, 0.67)	<0.001
**Diabetes, yes versus no**	1.91 (1.44, 2.53)	<0.001	1.14 (1.08, 1.20)	<0.001	1.65 (1.48, 1.85)	<0.001	1.25 (1.03, 1.51)	0.03	1.55 (1.39, 1.72)	<0.001
**Mean dosage** * **, per 10-mg/d increase**	1.04 (1.02, 1.05)	<0.001	1.02 (1.01, 1.03)	<0.001	1.03 (1.02, 1.04)	<0.001	1.03 (1.02, 1.05)	<0.001	1.04 (1.03, 1.04)	<0.001
**Other immunosuppressant, yes versus no**	0.75 (0.45, 1.23)	0.26	0.72 (0.65, 0.78)	<0.001	0.95 (0.80, 1.13)	0.57	1.40 (1.11, 1.76)	0.004	0.82 (0.69, 0.97)	0.02

*Prednisone equivalent.

CTD, connective tissue disease; IBD, inflammatory bowel disease; PMR/GCA, polymyalgia rheumatica/giant cell arteritis; RA, rheumatoid arthritis.

### Biological Risk Factors for Infection in Those Exposed to Glucocorticoids

Both lymphocyte count and albumin level were available before the infectious event (or a randomly selected date for those with no infectious outcome) in 34,401 (12.5%) out of the 275,072 glucocorticoid-exposed individuals. These patients were older, more frequently had polymyalgia rheumatica/giant cell arteritis or cancer, and received a higher dosage of glucocorticoids than those with no lymphocyte count and albumin level available (S2 Table). When analyzing the data obtained in this population, we found that the risk of all infections but herpes zoster decreased as the albumin level increased. On the other hand, the risk of infection was not associated with lymphocyte count, except for a moderate decrease in risk of cutaneous cellulitis in those with higher lymphocyte count (Table 4).

**Table 4:** Risk factors for infection in the population exposed to systemic glucocorticoids with data on albumin level and lymphocyte count (*n* = 34,401).

Variable	Infectious Outcome									
	Septicemia (*n* = 112)		LRTI (*n* = 2,530)		Cutaneous Cellulitis (*n* = 668)		Herpes Zoster (*n* = 253)		Local Candidiasis (*n* = 779)	
	HR (95% CI)	*p*-Value	HR (95% CI)	*p*-Value	HR (95% CI)	*p*-Value	HR (95% CI)	*p*-Value	HR (95% CI)	*p*-Value
**Age, per 10-y increase**	1.14 (0.98, 1.33)	0.08	1.05 (1.02, 1.08)	0.002	1.29 (1.21, 1.38)	<0.001	1.00 (0.91, 1.10)	0.97	0.87 (0.82, 0.91)	<0.001
**Gender, women versus men**	0.77 (0.53, 1.13)	0.19	1.01 (0.93, 1.10)	0.80	1.28 (1.09, 1.51)	0.003	1.04 (0.80, 1.35)	0.77	1.50 (1.29, 1.74)	<0.001
**Underlying disease**										
Asthma	1	—	1	—	1	—	1	—	1	—
COPD	2.00 (0.61, 6.52)	0.25	1.20 (1.03, 1.40)	0.02	1.00 (0.68, 1.47)	0.99	2.10 (1.00, 4.39)	0.05	1.00 (0.71, 1.39)	0.98
RA	0.47 (0.08, 2.61)	0.39	0.43 (0.36, 0.52)	<0.001	0.87 (0.60, 1.26)	0.46	1.47 (0.73, 2.98)	0.28	0.41 (0.28, 0.61)	<0.001
IBD	2.21 (0.49, 9.98)	0.30	0.20 (0.13, 0.29)	<0.001	0.63 (0.32, 1.25)	0.19	0.64 (0.18, 2.30)	0.49	0.64 (0.41, 1.01)	0.05
PMR/GCA	0.85 (0.28, 2.63)	0.78	0.25 (0.22, 0.29)	<0.001	0.52 (0.37, 0.72)	<0.001	1.50 (0.78, 2.87)	0.22	0.41 (0.31, 0.56)	<0.001
Cancer	5.17 (1.82, 14.65)	0.002	0.86 (0.74, 0.99)	0.04	1.01 (0.71, 1.45)	0.96	2.08 (1.02, 4.24)	0.04	2.04 (1.56, 2.65)	<0.001
CTD	2.76 (0.73, 10.50)	0.14	0.36 (0.27, 0.47)	<0.001	0.87 (0.54, 1.42)	0.58	1.94 (0.88, 4.24)	0.10	0.65 (0.42, 1.00)	0.05
Other	1.93 (0.68, 5.45)	0.22	0.49 (0.43, 0.56)	<0.001	1.01 (0.74, 1.37)	0.97	1.62 (0.86, 3.06)	0.14	0.69 (0.53, 0.89)	<0.001
**Diabetes, yes versus no**	1.49 (0.96, 2.32)	0.08	1.01 (0.91, 1.12)	0.88	1.43 (1.19, 1.72)	<0.001	1.03 (0.73, 1.44)	0.87	1.30 (1.08, 1.56)	0.006
**Mean dosage** * **, per 10-mg/d increase**	1.09 (1.04, 1.14)	<0.001	1.06 (1.04, 1.08)	<0.001	1.07 (1.04, 1.12)	<0.001	1.06 (1.00, 1.13)	0.04	1.09 (1.07, 1.10)	<0.001
**Other immunosuppressant, yes versus no**	0.52 (0.23, 1.18)	0.12	0.83 (0.72, 0.95)	0.006	0.95 (0.75, 1.20)	0.64	1.64 (1.17, 2.29)	0.004	0.77 (0.60, 0.99)	0.04
**Albumin level, per 1-g/l increase**	0.93 (0.90, 0.96)	<0.001	0.98 (0.97, 0.98)	<0.001	0.96 (0.94, 0.97)	<0.001	1.00 (0.97, 1.02)	0.85	0.96 (0.95, 0.97)	<0.001
**Lymphocyte count, per 1,000/mm** ^**3**^ **increase**	0.96 (0.90, 1.02)	0.19	1.00 (0.99, 1.01)	0.96	0.98 (0.95, 1.00)	0.03	0.99 (0.97, 1.02)	0.69	0.99 (0.98, 1.01)	0.37

*Prednisone equivalent.

CTD, connective tissue disease; IBD, inflammatory bowel disease; PMR/GCA, polymyalgia rheumatica/giant cell arteritis; RA, rheumatoid arthritis.

### Sensitivity Analyses

In order to ensure that the relative risk variations over duration of exposure observed for LRTI and local candidiasis (Fig 4) were not related to the high prevalence of people with asthma and COPD in our study population (as they could be at higher risk of LRTI and thrush on account of their underlying disease and their use of inhaled glucocorticoids), we ran a separate analysis on all patients excluding those with asthma or COPD (S1 Fig). We found that the relative risks over duration of exposure were similar to those of the overall population.

We also assessed risk factors of infection in those exposed to glucocorticoids after excluding all infections recorded within 1 mo after glucocorticoid initiation, rather than 15 d. Once again, the results were similar (S3 Table).

Lastly, we sought to assess the impact of potential confounders on the risk of some infectious outcomes. For instance, we hypothesized that the high observed risk of LRTI in patients with asthma or COPD could be associated with the severity of the underlying disease (the severity being associated with both the risk of LRTI and the risk of glucocorticoid prescription, and therefore being an unmeasured confounder). Assuming that 40% to 60% of those with asthma/COPD had a more severe condition that increased the risk of LRTI independently of glucocorticoid exposure (corresponding to a confounder prevalence in the overall study population of approximately 20% to 30%), the Schneeweiss rule-out approach analysis suggested that the severity of asthma/COPD would have to be very strongly associated with both glucocorticoid exposure and LRTI to fully explain the observed result (S2 Fig).

Because we found that the risk of septicemia was very strongly associated with the underlying disease cancer, we also assessed the impact of concomitant chemotherapy prescription in those with cancer (which can be associated with the risk of both septicemia and glucocorticoid prescription) on the risk of septicemia. As it was difficult to find estimates of the proportion of cancer patients prescribed chemotherapy, we carried out analyses assuming that 25%, 50%, and 75% of the individuals with cancer had chemotherapy (corresponding to a confounder prevalence in the overall study population of approximately 5%, 10%, and 15%, respectively). The analysis suggested that chemotherapy would have to be strongly associated with glucocorticoid exposure and septicemia to fully explain the observed results (S3 Fig).

## Discussion

In this population-based cohort study, we found that the risk of some bacterial, viral, and fungal infections was 2- to 6-fold higher in people prescribed oral glucocorticoids than in people matched for age, gender, and the underlying disease. We did not demonstrate an increase in risk for varicella, dermatophytosis, or scabies in the glucocorticoid-treated population, after adjustment for the underlying condition. The relative risk of infection depended largely on the type of infection. The relative increase in risk for LRTI and local candidiasis was highest during the first weeks of exposure and markedly decreased thereafter. The risk of infection increased with age and was higher in those with diabetes, in those prescribed a higher dosage of glucocorticoid, and in those with lower plasma albumin level. It also markedly depended on the underlying disease.

Many studies have reported on the impact of systemic glucocorticoid exposure on the occurrence of specific mycobacterial, fungal, or parasitic opportunistic infections in specific glucocorticoid-treated populations [4–6,9,10]. Other studies have focused on the risk of more common infections (mostly bacterial infections) in specific populations. In the study by Durand and Thomas, the adjusted rate ratios for LRTI, upper urinary tract infection, and sepsis were 1.48 (95% CI 1.34–1.65), 1.27 (95% CI 1.10–1.46), and 1.63 (95% CI 0.78–3.40), respectively, in 1,664 patients exposed to systemic glucocorticoids for giant cell arteritis compared to 8,078 matched control patients [17]. In a study of patients with systemic lupus erythematosus, the risk of post-surgical pneumonia (odds ratio [OR] 3.59 [95% CI 2.44–5.30]) and septicemia (OR 4.23 [95% CI 2.92–6.13]) was significantly increased in those previously exposed to systemic glucocorticoids [7]. It is unclear whether systemic glucocorticoids increase the risk of non-serious infections in rheumatoid arthritis patients. In a large case control study of more than 16,000 patients with rheumatoid arthritis published in 2011, the risk of non-serious infections was higher in those on systemic glucocorticoids (adjusted relative risk [RR] 1.10 [95% CI 1.04–1.16] for those exposed to 5–10 mg/d and 1.85 [95% CI 1.68–2.05] for those exposed to ≥20 mg/d) than in the unexposed group [18]. On the other hand, a meta-analysis of RCTs published the same year showed no increase in the risk of non-serious infections (RR 1.05 [95% CI 0.89–1.24] when the RCTs considered to report predominantly these events were analyzed) [19]. In contrast, the risk of common serious infections (e.g., bacterial pneumonia, herpes zoster, and skin or soft tissue infections) increased in these patients [19,20]. In patients with inflammatory bowel disease, the risk of serious common bacterial infections was increased by four times in those exposed to systemic glucocorticoids (adjusted RR 4.0 [95% CI 2.5–6.6]) [4]. Regarding viral infections, glucocorticoids increase the risk of viral reactivation (e.g., hepatitis B virus and herpes zoster) [21,22]. Whether or not viral infections are more common or severe in those exposed to glucocorticoids is still unclear [23–27]. There are numerous case reports of severe scabies (i.e., Norwegian) or dermatophytosis (i.e., deep dermatophyte infection) after exposure to glucocorticoids [28–32]. However, an increase in risk of common presentations of these infections in people exposed to glucocorticoids has never been demonstrated.

Regarding our results, we found that the risk of local candidiasis was much higher than that of bacterial, viral, or parasitic infections, with the exception of LRTI. There are many glucocorticoid-induced biological effects that result in increased susceptibility to fungal infections [8]. These include direct or indirect effects on lymphocytes, neutrophils, or eosinophils, but also direct effects on candida growth, morphogenesis, and virulence [8,33,34]. We also found that the risk of LRTI was much higher than the risk of other bacterial infections such as septicemia or cutaneous cellulitis. A significantly increased risk of pneumonia or recurrent pneumonia has previously been demonstrated with inhaled glucocorticoids in patients with asthma or COPD [35–37]. We further found that the risk of infection was associated with the underlying disease. Overall, patients with polymyalgia rheumatica/giant cell arteritis were at lower risk. This finding cannot be easily explained but is in accordance with previous studies demonstrating a low risk of bacterial or viral infection in these patients [17,38]. Lastly, we found that for LRTI and local candidiasis, the relative increase in risk was highest during the first weeks of exposure and markedly decreased thereafter. Whether the relative risk of infection induced by systemic glucocorticoids is more related to the treatment daily dosage or to the treatment duration is still unknown and debated. A meta-analysis showed that the risk was highest during the first days/weeks of therapy than for longer exposure [3]. In the large study by Dixon et al. [39], the risk of serious infection was higher in patients exposed for 1 mo to 30 mg/d of prednisolone equivalent (adjusted OR 1.84 [95% CI 1.58–4.00]) than in those exposed for 6 mo to 5 mg/d (adjusted OR 1.46 [95% CI 1.31–1.65]).

Biologically, a low albumin level was strongly associated with the risk of infection. The low albumin level could be indirectly (i.e., by being a marker of the malnutrition–inflammation syndrome) or directly (i.e., as an etiological factor) responsible for this higher risk of infection. For instance, a low albumin level is associated with a higher free glucocorticoid fraction, i.e., the biologically relevant fraction of the drug interacting with the glucocorticoid receptor [40]. A correlation between increased free fraction of the drug due to low albumin concentration and glucocorticoid adverse effects has previously been evidenced during prednisone therapy [41].

Our study has several strengths. We used a large population-based sample of patients of both sexes, across all adult age groups, and with many underlying diseases and comorbidities. This enabled us to compare the impact of systemic glucocorticoids on different bacterial, viral, fungal, and parasitic infections using the same method of assessment. Moreover, the study population is an unselected population of patients exposed to systemic glucocorticoids in primary care; to our knowledge, such a study has never been done previously.

However, there are also some limitations to our study. First, the use of anonymized electronic medical records and reliance on diagnostic codes could have resulted in some degree of misclassification of the infectious outcomes. However, we chose to study infections currently seen in primary care. Second, a reporting bias is possible, as general practitioners could be more prone to record an infection in those exposed to glucocorticoids. However, very different relative risks were observed for the studied infections, and we believe that it is unlikely that general practitioners would more systematically record local candidiasis than herpes zoster or septicemia in those prescribed glucocorticoids. Further, such bias is unlikely to occur for severe events such as septicemia. Third, we chose to study only events for which a precise diagnostic code was recorded. For instance, for LRTI, we did not include events recorded as “chest infections,” and we included only bacterial cutaneous infections that were recorded as “cutaneous cellulitis” or “erysipelas,” not those reported as “bacterial skin or soft tissue infection.” Hence, the proportions experiencing the outcomes of interest as reported in Table 2 are likely to be much lower than what really occurred. For this reason, we chose not to assess the incidence rates of the infections of interest in the study population, as they would be underestimates of the true incidence rates. Fourth, we did not observe an increase in the risk of scabies or varicella in glucocorticoid-exposed patients, but the number of records of these events was small and statistical power was therefore limited. Fifth, no data were available regarding adherence to glucocorticoid treatment. We found that those exposed to other immunosuppressants in addition to glucocorticoids may be less likely to develop infections (with the exception of herpes zoster), but this finding does not exclude the possibility that those whose conditions are well-controlled with other immunosuppressants may be less adherent to glucocorticoids, which are perceived by patients as a dangerous drug [42]. Sixth, even though we tried to assess the impact of the most obvious potential unmeasured confounders (disease severity for those with asthma/COPD and use of concomitant chemotherapy for those with cancer), it is likely that some other confounders were not accounted for in the analyses. We cannot tell whether adjusting for these unmeasured confounders would have radically changed the findings. Seventh, for LRTI and local candidiasis, the relative decrease in risk over the duration of exposure can be explained, at least partly, by the fact that the most at-risk patients may have developed the infectious condition during the first weeks or months of glucocorticoid exposure, which could have led to an early cessation of glucocorticoids. Therefore, those who are still exposed to glucocorticoids after many months may be patients who are inherently at low risk of LRTI or local candidiasis. Similarly, it can be assumed that there are some clinical factors that influence general practitioners in their decision to prescribe more or less time on glucocorticoids (e.g., severity of the underlying disease, general health state of the patient). If these factors are associated with the risk of infection, they could contribute to the patterns of time-dependent risks that were observed in our study. However, if these were important cofounders, we believe that the same time-dependent patterns would have been observed for all assessed outcomes. Eight, while the duration of a prescription can easily be defined for short-term glucocorticoid exposures, some may argue that duration may be less precise for long-term exposures. However, even for long-term prescriptions, treatment renewal is usually made every 1 to 3 mo, which allowed us to estimate fairly precisely when treatment ended, with an uncertainty margin of no more than a few days. Lastly, some treatments, such as chemotherapy or use of biologicals prescribed in hospital, were not taken into account in the analyses as they are not prescribed in primary care and are therefore not recorded in THIN.

Our estimates have several practical implications. First, patients prescribed systemic glucocorticoids are at very high risk of LRTI. This should be considered when prescribing systemic glucocorticoids, in particular in those with asthma or COPD [43], and when deciding which patients might need more intensive follow-up and immunization [44]. Further, a skin and mucous examination is probably advisable in patients exposed to systemic glucocorticoid therapy for more than a few days so as to screen out (and treat) both candidiasis and foot mycosis (as a risk factor for cutaneous cellulitis). Lastly, hypoalbuminemia seems to be a biological factor as important as diabetes in increasing the risk of infections in glucocorticoid-exposed patients. While fasting glycemia is often monitored in these patients, screening for albumin level is not currently recommended at baseline and during glucocorticoid exposure [45].

## Supporting Information

S1 Fig Incidence rate ratios of infections according to duration of glucocorticoid exposure in a selected population excluding individuals with asthma or COPD. (TIF)

S2 Fig Severity of asthma/COPD as a potential confounder. As outlined by Schneeweiss [15], each line splits the area into two. The upper right area represents all parameter combinations of OR_ec_ (association between drug use category and confounder) and RR_cd_ (association between confounder and disease outcome) that would create confounding by an unmeasured factor strong enough to move the point estimate from the apparent relative risk (here 5.84) to the null (i.e., RR = 1) or even lower, i.e., to make the association go away. Conversely, the area to the lower left represents all parameter combinations that would not be able to move the apparent relative risk to the null. Here, we assumed a prevalence of the confounder (*P*_c_) in the study population of 0.20, 0.25, and 0.30.
(TIF)

S3 Fig Concomitant prescription of chemotherapy in those with cancer as potential confounder. In this example, the apparent relative risk is 2.12, and we carried out analyses assuming that prevalence of the confounder (*P*_c_) in the overall study population was 0.05, 0.10, and 0.15.
(TIF)

S1 Table Code lists used to define infectious outcomes. (XLSX)

S2 Table Comparison of individuals with and without available lymphocyte count and albumin level before glucocorticoid initiation. (DOCX)

S3 Table Risk factors for infection in the overall population exposed to systemic glucocorticoids excluding all infectious events recorded during the first month after glucocorticoid initiation. (DOCX)

S1 Text STROBE Statement—checklist of items that should be included in reports of observational studies. (DOCX)

## Abbreviations

COPD: chronic obstructive pulmonary disease
IQR: interquartile range
LRTI: lower respiratory tract infection
OR: odds ratio
RCT: randomized controlled trial
RR: relative risk
THIN: The Health Improvement Network

## References

[1] Fardet L, Petersen I, Nazareth I. Prevalence of long-term oral glucocorticoid prescriptions in the UK over the past 20 years. Rheumatology (Oxford). 2011;50:1982–1990. 21393338 10.1093/rheumatology/ker017

[2] Overman RA, Yeh J-Y, Deal CL. Prevalence of oral glucocorticoid usage in the United States: a general population perspective. Arthritis Care Res. 2013;65:294–298. 10.1002/acr.21796 22807233

[3] Stuck AE, Minder CE, Frey FJ. Risk of infectious complications in patients taking glucocorticosteroids. Rev Infect Dis. 1989;11:954–963. 2690289 10.1093/clinids/11.6.954

[4] Brassard P, Bitton A, Suissa A, Sinyavskaya L, Patenaude V, Suissa S. Oral corticosteroids and the risk of serious infections in patients with elderly-onset inflammatory bowel diseases. Am J Gastroenterol. 2014;109:1795–1802. 10.1038/ajg.2014.313 25267328

[5] Chang YS, Liu CJ, Ou SM, Hu YW, Chen TJ, Lee HT, et al Tuberculosis infection in primary Sjögren’s syndrome: a nationwide population-based study. Clin Rheumatol. 2014;33:377–383. 10.1007/s10067-013-2408-y 24170112

[6] Fernàndez-Sabé N, Cervera C, López-Medrano F, Llano M, Sáez E, Len O, et al Risk factors, clinical features, and outcomes of listeriosis in solid-organ transplant recipients: a matched case-control study. Clin Infect Dis. 2009;49:1153–1159. 10.1086/605637 19751149

[7] Lin JA, Liao CC, Lee YJ, Wu CH, Huang WQ, Chen TL. Adverse outcomes after major surgery in patients with systemic lupus erythematosus: a nationwide population-based study. Ann Rheum Dis. 2014;73:1646–1651. 10.1136/annrheumdis-2012-202758 23740232

[8] Lionakis MS, Kontoyiannis DP. Glucocorticoids and invasive fungal infections. Lancet. 2003;362:1828–1838. 14654323 10.1016/S0140-6736(03)14904-5

[9] O’Donnell MR, Schmidt GM, Tegtmeier BR, Faucett C, Fahey JL, Ito J, et al Prediction of systemic fungal infection in allogeneic marrow recipients: impact of amphotericin prophylaxis in high-risk patients. J Clin Oncol. 1994;12:827–834. 8151325 10.1200/JCO.1994.12.4.827

[10] Peleg AY, Husain S, Qureshi ZA, Silveira FP, Sarumi M, Shutt KA, et al Risk factors, clinical characteristics, and outcome of Nocardia infection in organ transplant recipients: a matched case-control study. Clin Infect Dis. 2007;44:1307–1314. 17443467 10.1086/514340

[11] Blak BT, Thompson M, Dattani H, Bourke A. Generalisability of The Health Improvement Network (THIN) database: demographics, chronic disease prevalence and mortality rates. Inform Prim Care. 2011;19:251–255. 22828580 10.14236/jhi.v19i4.820

[12] Lewis JD, Schinnar R, Bilker WB, Wang X, Strom BL. Validation studies of The Health Improvement Network (THIN) database for pharmacoepidemiology research. Pharmacoepidemiol Drug Saf. 2007;16:393–401. 17066486 10.1002/pds.1335

[13] MedicinesComplete. British National Formulary. 2016 [cited 1 Apr 2016]. London: Pharmaceutical Press.

[14] Chisholm J. The Read clinical classification. BMJ. 1990;300:1092 2344534 10.1136/bmj.300.6732.1092 PMC1662793

[15] Schneeweiss S. Sensitivity analysis and external adjustment for unmeasured confounders in epidemiologic database studies of therapeutics. Pharmacoepidemiol Drug Saf. 2006;15:291–303. 16447304 10.1002/pds.1200

[16] Division of Pharamacoepidemiology and Pharmacoeconomics. Sensitivity analysis of confounding: April 20, 2008. Boston: Brigham and Women’s Hospital. 2008 Apr 20 [cited 16 Nov 2015]. Available: http://www.drugepi.org/dope-downloads/#Sensitivity%20Analysis.

[17] Durand M, Thomas SL. Incidence of infections in patients with giant cell arteritis: a cohort study. Arthritis Care Res. 2012;64:581–588. 10.1002/acr.21569 22184094

[18] Durand M, Dixon WG, Kezouh A, Bernatsky S, Suissa S. The influence of systemic glucocorticoid therapy upon the risk of non-serious infection in older patients with rheumatoid arthritis: a nested case-control study. Ann Rheum Dis. 2011;70:956–960. 10.1136/ard.2010.144741 21285116 PMC3086054

[19] Dixon WG, Suissa S, Hudson M. The association between systemic glucocorticoid therapy and the risk of infection in patients with rheumatoid arthritis: systematic review and meta-analyses. Arthritis Res Ther. 2011;13:R139 10.1186/ar3453 21884589 PMC3239382

[20] Widdifield J, Bernatsky S, Paterson JM, Gunraj N, Thorne JC, Pope J, et al Serious infections in a population-based cohort of 86,039 seniors with rheumatoid arthritis. Arthritis Care Res. 2013;65:353–361. 10.1002/acr.21812 22833532

[21] Vento S, Cainelli F, Longhi MS. Reactivation of replication of hepatitis B and C viruses after immunosuppressive therapy: an unresolved issue. Lancet Oncol. 2002;3:333–340. 12107020 10.1016/s1470-2045(02)00773-8

[22] Smitten AL, Choi HK, Hochberg MC, Suissa S, Simon TA, Testa MA, et al The risk of herpes zoster in patients with rheumatoid arthritis in the United States and the United Kingdom. Arthritis Rheum. 2007; 57:1431–1438. 18050184 10.1002/art.23112

[23] Blumentals WA, Arreglado A, Napalkov P, Toovey S. Rheumatoid arthritis and the incidence of influenza and influenza-related complications: a retrospective cohort study. BMC Musculoskelet Disord. 2012; 13:158 10.1186/1471-2474-13-158 22925480 PMC3495205

[24] Wu UI, Wang JT, Ho YC, Pan SC, Chen YC, Chang SC. Factors associated with development of complications among adults with influenza: a 3-year prospective analysis. J Formos Med Assoc. 2012;111:364–369. 10.1016/j.jfma.2011.04.005 22817813

[25] Myles P, Nguyen-Van-Tam JS, Semple MG, Brett SJ, Bannister B, Read RC, et al Differences between asthmatics and nonasthmatics hospitalised with influenza A infection. Eur Respir J. 2013;41:824–831. 10.1183/09031936.00015512 22903963 PMC3612580

[26] Hill G, Chauvenet AR, Lovato J, McLean TW. Recent steroid therapy increases severity of varicella infections in children with acute lymphoblastic leukemia. Pediatrics. 2005;116:e525–9. 16199681 10.1542/peds.2005-0219

[27] Dowell SF, Bresee JS. Severe varicella associated with steroid use. Pediatrics. 1993;92:223–228. 8337020

[28] Jacobs PH. Majocchi’s granuloma (due to therapy with steroid and occlusion). Cutis. 1986;38:23 3731863

[29] Li FQ, Lv S, Xia JX. Majocchi’s granuloma after topical corticosteroids therapy. Case Rep Dermatol Med. 2014:507176 10.1155/2014/507176 25405039 PMC4227411

[30] Jaramillo-Ayerbe F, Berrío-Muñoz J. Ivermectin for crusted Norwegian scabies induced by use of topical steroids. Arch Dermatol. 1998;134:143–145. 9487205 10.1001/archderm.134.2.143

[31] Lipitz R, Tur E, Brenner S, Krakowski A. Norwegian scabies following topical corticosteroid therapy. Isr J Med Sci. 1981;17:1165–1168. 7327916

[32] Timpatanapong P, Tasanapradit P, Indulak S, Sundaravej P. Norwegian scabies in acute SLE patient treated with high dose corticosteroid. J Med Assoc Thai. 1974;57:514–516. 4443662

[33] Chen SC-A, Lewis RE, Kontoyiannis DP. Direct effects of non-antifungal agents used in cancer chemotherapy and organ transplantation on the development and virulence of Candida and Aspergillus species. Virulence. 2011;2:280–295. 21701255 10.4161/viru.2.4.16764 PMC3173675

[34] Ghannoum MA, Elteen KA. Effect of growth of Candida spp. in the presence of various glucocorticoids on the adherence to human buccal epithelial cells. Mycopathologia. 1987;98:171–178. 3587341 10.1007/BF00437652

[35] Drummond MB, Dasenbrook EC, Pitz MW, Murphy DJ, Fan E. Inhaled corticosteroids in patients with stable chronic obstructive pulmonary disease: a systematic review and meta-analysis. JAMA. 2008;300:2407–2416. 10.1001/jama.2008.717 19033591 PMC4804462

[36] Eurich DT, Lee C, Marrie TJ, Majumdar SR. Inhaled corticosteroids and risk of recurrent pneumonia: a population-based, nested case-control study. Clin Infect Dis. 2013;57:1138–1144. 10.1093/cid/cit472 23872948

[37] McKeever T, Harrison TW, Hubbard R, Shaw D. Inhaled corticosteroids and the risk of pneumonia in people with asthma: a case-control study. Chest. 2013;144:1788–1794. 10.1378/chest.13-0871 23990003

[38] Schäfer VS, Kermani TA, Crowson CS, Hunder GG, Gabriel SE, Ytterberg SR, et al Incidence of herpes zoster in patients with giant cell arteritis: a population-based cohort study. Rheumatology (Oxford). 2010; 49:2104–2108. 20627970 10.1093/rheumatology/keq200 PMC2981027

[39] Dixon WG, Abrahamowicz M, Beauchamp ME, Ray DW, Bernatsky S, Suissa S, et al Immediate and delayed impact of oral glucocorticoid therapy on risk of serious infection in older patients with rheumatoid arthritis: a nested case-control analysis. Ann Rheum Dis. 2012;71:1128–1133. 10.1136/annrheumdis-2011-200702 22241902 PMC3375584

[40] Czock D, Keller F, Rasche FM, Häussler U. Pharmacokinetics and pharmacodynamics of systemically administered glucocorticoids. Clin Pharmacokinet. 2005;44:61–98. 15634032 10.2165/00003088-200544010-00003

[41] Lewis GP, Jusko WJ, Graves L, Burke CW. Prednisone side-effects and serum-protein levels. A collaborative study. Lancet. 1971;2:778–780. 4106604 10.1016/s0140-6736(71)92738-3

[42] Cullen G, Kelly E, Murray FE. Patients’ knowledge of adverse reactions to current medications. Br J Clin Pharmacol. 2006;62:232–236. 16842399 10.1111/j.1365-2125.2006.02642.x PMC1885086

[43] National Institute for Health and Care Excellence. Chronic obstructive pulmonary disease in over 16s: diagnosis and management. NICE guidelines [CG101]. 2010 June [cited 1 May 2015]. Available: https://www.nice.org.uk/guidance/cg101/. 31211541

[44] Fardet L, Petersen I, Nazareth I. Monitoring of patients on long-term glucocorticoid therapy: a population-based cohort study. Medicine (Baltimore). 2015;94:e647. 25881838 10.1097/MD.0000000000000647 PMC4602514

[45] Duru N, van der Goes MC, Jacobs JW, Andrews T, Boers M, Buttgereit F, et al EULAR evidence-based and consensus-based recommendations on the management of medium to high-dose glucocorticoid therapy in rheumatic diseases. Ann Rheum Dis. 2013;72:1905–1913. 10.1136/annrheumdis-2013-203249 23873876

