# Supplementary figures and images for: Common Infections in Patients Prescribed Systemic Glucocorticoids in Primary Care: A Population-Based Cohort Study
Source: PLoS Med. 2016 May 24;13(5):e1002024. doi: 10.1371/journal.pmed.1002024 (PMC4878789; doi:10.1371/journal.pmed.1002024)

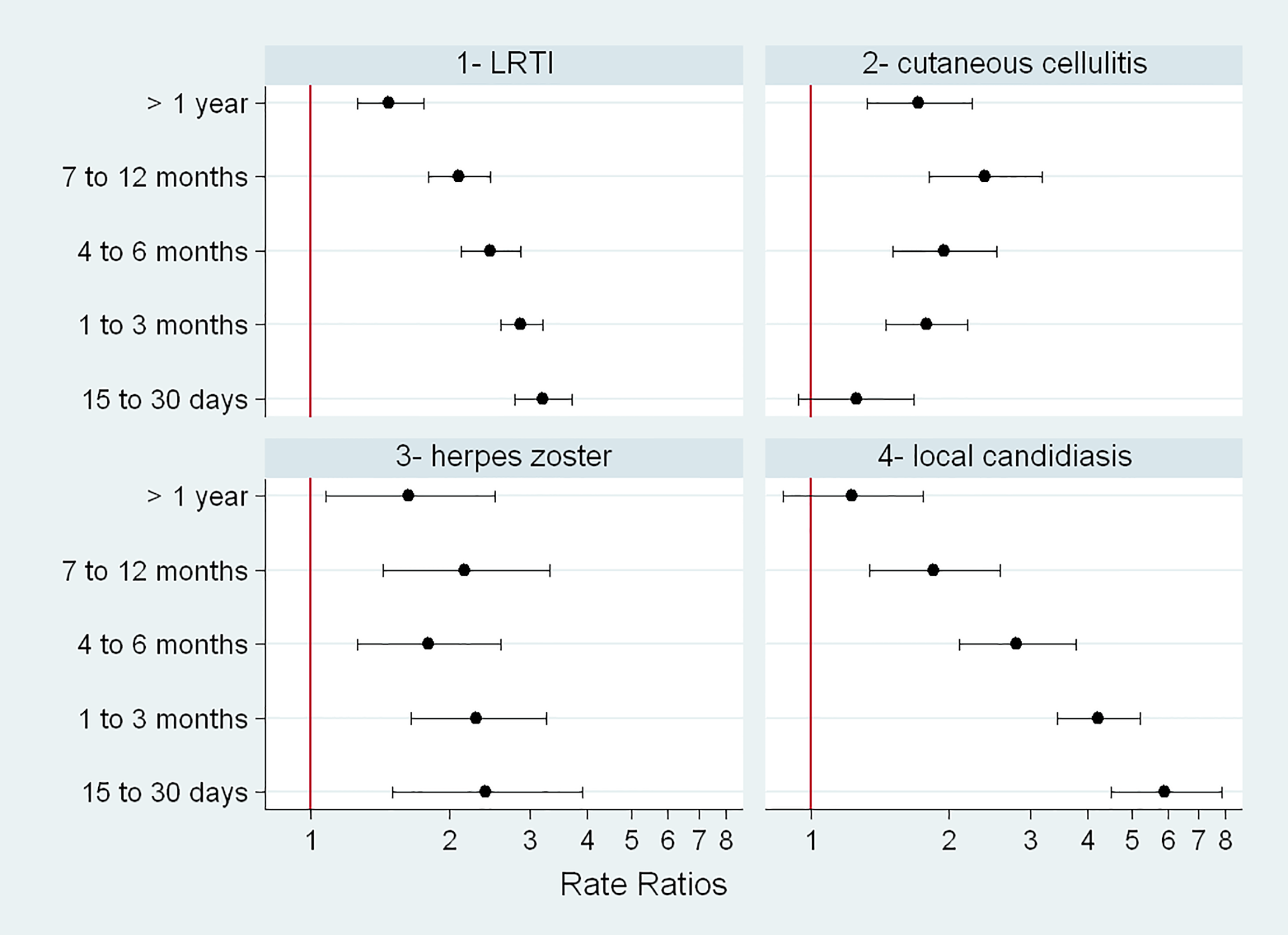

Supplement: S1 Fig — (TIF) [file pmed.1002024.s001.tif]

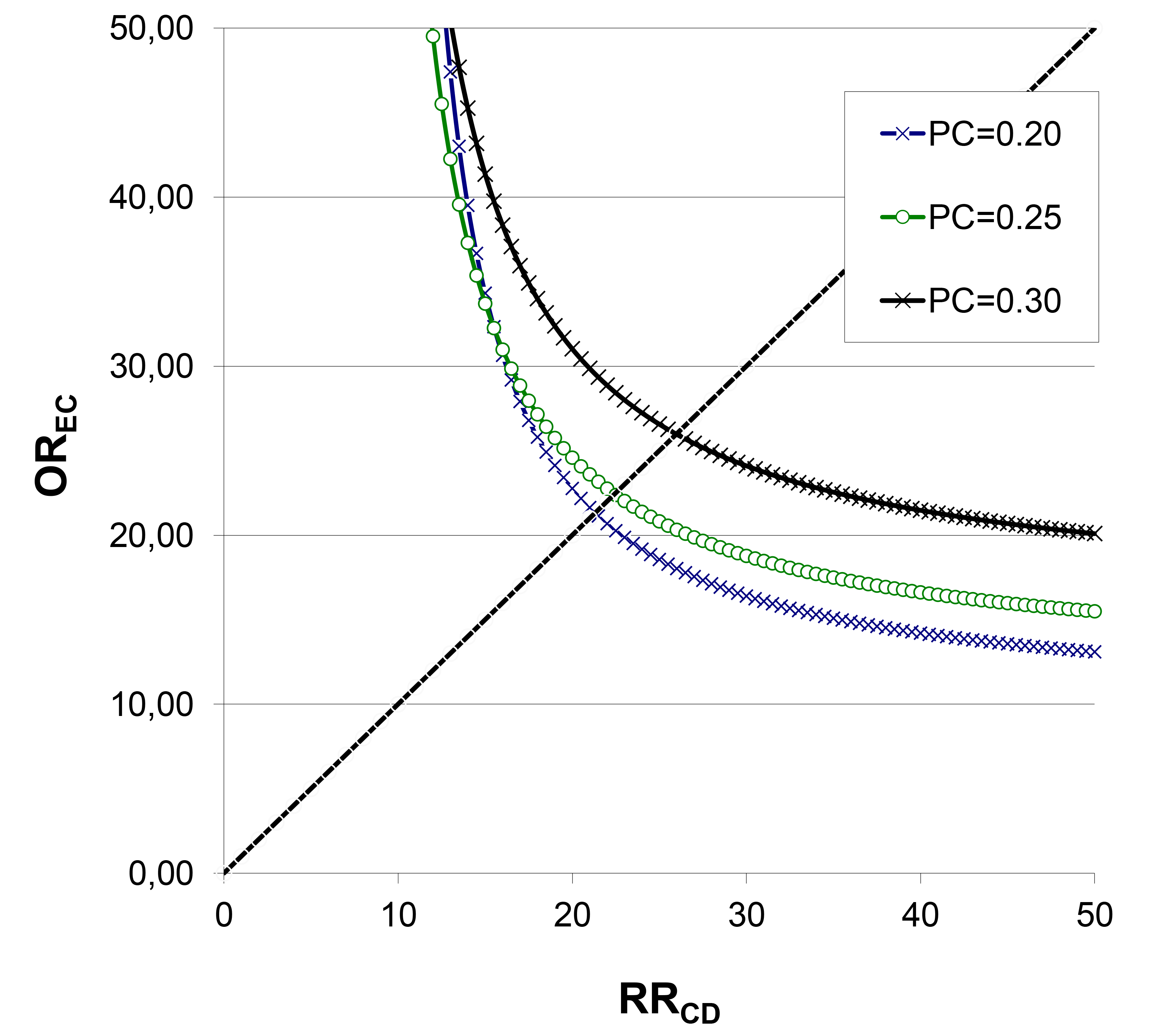

Supplement: S2 Fig — As outlined by Schneeweiss [15], each line splits the area into two. The upper right area represents all parameter combinations of ORec (association between drug use category and confounder) and RRcd (association between confounder and disease outcome) that would create confounding by an unmeasured factor strong enough to move the point estimate from the apparent relative risk (here 5.84) to the null (i.e., RR = 1) or even lower, i.e., to make the association go away. Conversely, the area to the lower left represents all parameter combinations that would not be able to move the apparent relative risk to the null. Here, we assumed a prevalence of the confounder (P c) in the study population of 0.20, 0.25, and 0.30. (TIF) [file pmed.1002024.s002.tif]

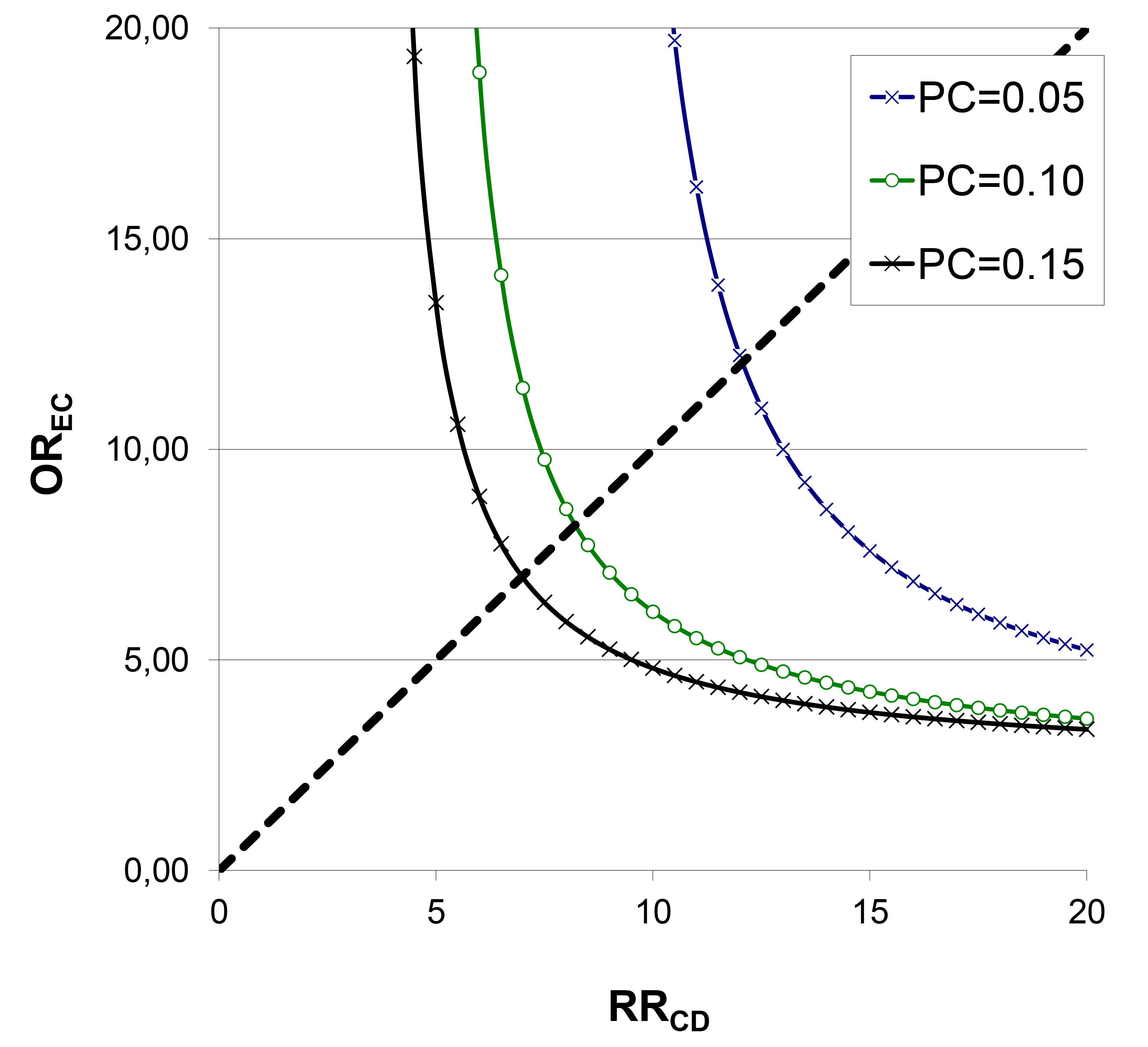

Supplement: S3 Fig — In this example, the apparent relative risk is 2.12, and we carried out analyses assuming that prevalence of the confounder (P c) in the overall study population was 0.05, 0.10, and 0.15. (TIF) [file pmed.1002024.s003.tif]
