# Supplementary material for: Common Infections in Patients Prescribed Systemic Glucocorticoids in Primary Care: A Population-Based Cohort Study
Source: PLoS Med. 2016 May 24;13(5):e1002024. doi: 10.1371/journal.pmed.1002024 (PMC4878789; doi:10.1371/journal.pmed.1002024)
Supplement: S2 Table — (DOCX) [file pmed.1002024.s005.docx]

|  | **GC-exposed patients without lymphocyte count and albumin level available**  **N=240,671** | **GC-exposed patients with both lymphocyte count and albumin level available**  **N=34,401** |
| --- | --- | --- |
| **Female**, n (%) | 138783 (57.7) | 20216 (58.8) |
| **Age**, years | 62 [47-74] | 69 [57-78] |
| **Underlying diseases,** n (%)  Asthma  Chronic obstructive pulmonary disease  Cancer  Polymyalgia rheumatica/giant cell arteritis  Inflammatory bowel diseases  Rheumatoid arthritis  Connective tissue diseases  Other or unknown | 57240 (23.8)  31007 (12.9)  22014 (9.1)  18434 (7.6)  8185 (3.4)  5183 (2.2)  3318 (1.4)  95290 (39.6) | 4923 (14.3)  3988 (11.6)  4488 (13.0)  4820 (14.0)  1429 (4.2)  1823 (5.3)  774 (2.3)  12156 (35.3) |
| **Duration of GC exposure,** days | 33 [21-66] | 42 [25-100] |
| **Initial GC daily dosage,** mg | 15 [10-30] | 20 [10-30] |
| **Infectious events,** n (%) | 13793 (5.7) | 4505 (13.1) |

GC: glucocorticoid
