# Supplementary material for: Common Infections in Patients Prescribed Systemic Glucocorticoids in Primary Care: A Population-Based Cohort Study
Source: PLoS Med. 2016 May 24;13(5):e1002024. doi: 10.1371/journal.pmed.1002024 (PMC4878789; doi:10.1371/journal.pmed.1002024)
Supplement: S3 Table — (DOCX) [file pmed.1002024.s006.docx]

|  | **Septicemia (n=260)** | | **Lower respiratory tract**  **infection (n=7653)** | | **Cutaneous cellulitis**  **(n=1790)** | | **Herpes zoster (n=697)** | | **Local candidiasis**  **(n=1806)** | |
| --- | --- | --- | --- | --- | --- | --- | --- | --- | --- | --- |
|  | **HR (95%CI)** | **p** | **HR (95%CI)** | **p** | **HR (95%CI)** | **p** | **HR (95%CI)** | **p** | **HR (95%CI)** | **p** |
| **Age**, per 10 years increase | 1.11 (1.02, 1.22) | 0.02 | 1.10 (1.08, 1.12) | <0.001 | 1.38 (1.33, 1.43) | <0.001 | 1.07 (1.01, 1.13) | 0.02 | 0.91 (0.88, 0.94) | <0.001 |
| **Gender**, women versus men | 0.89 (0.69, 1.14) | 0.36 | 1.00 (0.95, 1.05) | 0.99 | 1.33 (1.20, 1.47) | <0.001 | 0.99 (0.85, 1.16) | 0.93 | 1.53 (1.38, 1.68) | <0.001 |
| **Underlying disease**  asthma  COPD  RA  IBD  PMR/GCA  Cancer  CTD  other | 1  3.28 (1.39, 7.78)  2.78 (1.09, 7.10)  2.99 (1.00, 8.95)  1.25 (0.54, 2.88)  11.81 (5.43, 25.68)  4.54 (1.71, 12.05)  3.18 (1.46, 6.89) | -  0.007  0.03  0.05  0.61  <0.001  0.002  0.003 | 1  1.22 (1.12, 1.31)  0.36 (0.32, 0.41)  0.21 (0.17, 0.26)  0.21 (0.19, 0.23)  0.71 (0.66, 0.78)  0.33 (0.28, 0.39)  0.37 (0.34, 0.39) | -  <0.001  <0.001  <0.001  <0.001  <0.001  <0.001  <0.001 | 1  1.06 (0.84, 1.32)  0.78 (0.61, 0.99)  0.71 (0.48, 1.07)  0.50 (0.41, 0.60)  1.04 (0.84, 1.29)  0.94 (0.69, 1.27)  0.75 (0.63, 0.90) | -  0.63  0.04  0.10  <0.001  0.72  0.69  0.002 | 1  0.86 (0.56, 1.32)  0.89 (0.59, 1.33)  0.67 (0.35, 1.26)  0.87 (0.63, 1.20)  1.51 (1.06, 2.15)  1.53 (1.00, 2.36)  0.95 (0.70, 1.30) | -  0.49  0.56  0.22  0.41  0.02  0.04  0.76 | 1  0.98 (0.80, 1.20)  0.45 (0.35, 0.59)  0.61 (0.46, 0.82)  0.34 (0.28, 0.41)  1.92 (1.22, 2.26)  0.70 (0.53, 0.92)  0.57 (0.49, 0.67) | -  0.85  <0.001  0.001  <0.001  <0.001  0.01  <0.001 |
| **Diabetes**, yes versus no | 2.06 (1.53, 2.76) | <0.001 | 1.13 (1.06, 1.21) | <0.001 | 1.60 (1.42, 1.81) | <0.001 | 1.24 (1.01, 1.53) | 0.04 | 1.55 (1.36, 1.76) | <0.001 |
| **Mean dosage***, per 10 mg/d increase | 1.03 (1.02, 1.05) | <0.001 | 1.02 (1.01, 1.03) | <0.001 | 1.02 (1.01, 1.03) | <0.001 | 1.03 (1.02, 1.05) | <0.001 | 1.03 (1.02, 1.04) | <0.001 |
| **Other immunosuppressant**, yes versus no | 0.80 (0.48, 1.32) | 0.37 | 0.77 (0.70, 0.85) | <0.001 | 1.00 (0.84, 1.21) | 0.93 | 1.41 (1.11, 1.79) | 0.005 | 0.82 (0.68, 0.98) | 0.03 |

COPD: chronic obstructive pulmonary disease, RA: rheumatoid arthritis, IBD: inflammatory bowel disease, PMR/GCA: polymyalgia rheumatica/giant cell arteritis, CTD: connective tissue disease

* of prednisone equivalent
