# Supplementary material for: Common Infections in Patients Prescribed Systemic Glucocorticoids in Primary Care: A Population-Based Cohort Study
Source: PLoS Med. 2016 May 24;13(5):e1002024. doi: 10.1371/journal.pmed.1002024 (PMC4878789; doi:10.1371/journal.pmed.1002024)
Supplement: S1 Text — (DOCX) [file pmed.1002024.s007.docx]

STROBE Statement—checklist of items that should be included in reports of observational studies

|  | Item No | Recommendation |  |
| --- | --- | --- | --- |
| **Title and abstract** | 1 | (*a*) Indicate the study’s design with a commonly used term in the title or the abstract | Done title and abstract |
|  |  | (*b*) Provide in the abstract an informative and balanced summary of what was done and what was found | Done |
| Introduction | | |  |
| Background/rationale | 2 | Explain the scientific background and rationale for the investigation being reported | Done first paragraph |
| Objectives | 3 | State specific objectives, including any prespecified hypotheses | Done second paragraph |
| Methods | | |  |
| Study design | 4 | Present key elements of study design early in the paper | Done Methods and Materials, paragraphs 2-6 |
| Setting | 5 | Describe the setting, locations, and relevant dates, including periods of recruitment, exposure, follow-up, and data collection | Done Methods and Materials, paragraph 2 |
| Participants | 6 | (*a*) *Cohort study*—Give the eligibility criteria, and the sources and methods of selection of participants. Describe methods of follow-up  *Case-control study*—Give the eligibility criteria, and the sources and methods of case ascertainment and control selection. Give the rationale for the choice of cases and controls | Done Methods and Materials, paragraphs 5-6 |
| Variables | 7 | Clearly define all outcomes, exposures, predictors, potential confounders, and effect modifiers. Give diagnostic criteria, if applicable | Done Methods and Materials, paragraphs 4 & 7 |
| Data sources/ measurement | 8* | For each variable of interest, give sources of data and details of methods of assessment (measurement). Describe comparability of assessment methods if there is more than one group | Done Methods and Materials, paragraphs 2-6 |
| Bias | 9 | Describe any efforts to address potential sources of bias | Done Methods and Materials, paragraph 8 and Discussion paragraph 6 |
| Study size | 10 | Explain how the study size was arrived at | N/A |
| Quantitative variables | 11 | Explain how quantitative variables were handled in the analyses. If applicable, describe which groupings were chosen and why | Done Methods and Materials, paragraph 7 |
| Statistical methods | 12 | (*a*) Describe all statistical methods, including those used to control for confounding | Done Methods and Materials, paragraph 8 |
|  |  | (*b*) Describe any methods used to examine subgroups and interactions | Done Methods and Materials, paragraph 8 |
|  |  | (*c*) Explain how missing data were addressed | N/A |
|  |  | (*d*) *Cohort study*—If applicable, explain how loss to follow-up was addressed  *Case-control study*—If applicable, explain how matching of cases and controls was addressed  *Cross-sectional study*—If applicable, describe analytical methods taking account of sampling strategy | N/A |
|  |  | (*e*) Describe any sensitivity analyses | Done Methods and Materials, paragraph 8 |

Continued on next page

| Results | | |  |
| --- | --- | --- | --- |
| Participants | 13* | (a) Report numbers of individuals at each stage of study—eg numbers potentially eligible, examined for eligibility, confirmed eligible, included in the study, completing follow-up, and analysed | Done Fig1 |
|  |  | (b) Give reasons for non-participation at each stage | N/A |
|  |  | (c) Consider use of a flow diagram | Done Fig1 |
| Descriptive data | 14* | (a) Give characteristics of study participants (eg demographic, clinical, social) and information on exposures and potential confounders | Done Table1 |
|  |  | (b) Indicate number of participants with missing data for each variable of interest | N/A |
|  |  | (c) *Cohort study*—Summarise follow-up time (eg, average and total amount) | Done Table 2 |
| Outcome data | 15* | *Cohort study*—Report numbers of outcome events or summary measures over time | Done Table2 |
|  |  | *Case-control study—*Report numbers in each exposure category, or summary measures of exposure | Done Table1 |
| Main results | 16 | (*a*) Give unadjusted estimates and, if applicable, confounder-adjusted estimates and their precision (eg, 95% confidence interval). Make clear which confounders were adjusted for and why they were included | Done Fig 2 |
|  |  | (*b*) Report category boundaries when continuous variables were categorized | N/A |
|  |  | (*c*) If relevant, consider translating estimates of relative risk into absolute risk for a meaningful time period | Not done (but proportions and total at-risk time provided in Table 2) |
| Other analyses | 17 | Report other analyses done—eg analyses of subgroups and interactions, and sensitivity analyses | Done Results section, paragraph 6 |
| Discussion | | |  |
| Key results | 18 | Summarise key results with reference to study objectives | Done first paragraph |
| Limitations | 19 | Discuss limitations of the study, taking into account sources of potential bias or imprecision. Discuss both direction and magnitude of any potential bias | Done sixth paragraph |
| Interpretation | 20 | Give a cautious overall interpretation of results considering objectives, limitations, multiplicity of analyses, results from similar studies, and other relevant evidence | Done paragraph 2-4 |
| Generalisability | 21 | Discuss the generalisability (external validity) of the study results | N/A |
| Other information | | |  |
| Funding | 22 | Give the source of funding and the role of the funders for the present study and, if applicable, for the original study on which the present article is based | No funding |
